# Identification of New *Fusarium sulawense* Strains Causing Soybean Pod Blight in China and Their Control Using Carbendazim, Dipicolinic Acid and Kojic Acid

**DOI:** 10.3390/ijerph191710531

**Published:** 2022-08-24

**Authors:** Qing Sun, Shi-Ling Zhang, Yong-Jing Xie, Mei-Ting Xu, Daniela D. Herrera-Balandrano, Xin Chen, Su-Yan Wang, Xin-Chi Shi, Pedro Laborda

**Affiliations:** School of Life Sciences, Nantong University, Nantong 226019, China

**Keywords:** *Fusarium* species, soybean diseases, carbendazim, antifungal activity, causal agent, mycotoxins

## Abstract

Soybean plants are highly susceptible to *Fusarium* species, which significantly reduce soybean production and quality. Several *Fusarium* species have been reported to synthesize mycotoxins, such as trichothecene, which have been related to major human diseases. In November 2021, soybean pods in Nantong municipality, China, showed black necrotic lesions during the harvest stage. The disease incidence reached 69%. The pathogen was identified as *Fusarium sulawense* via morphological analysis and sequencing of *ITS*, *EF1-α* and *RPB2* genes. A PCR assay with primers targeting the trichothecene biosynthesis genes suggested that the three isolates could synthesize trichothecenes. The effectiveness of fungicide carbendazim and natural metabolites dipicolinic acid and kojic acid was screened for the management of *F. sulawense* on postharvest soybean pods. The highest efficacy was obtained when combining 3.8 mg/mL carbendazim and 0.84 mg/mL dipicolinic acid (curative efficacy: 49.1% lesion length inhibition; preventive efficacy: 82.7% lesion length inhibition), or 1.9 mg/mL carbendazim and 0.71 mg/mL kojic acid (preventive efficacy: 84.9% lesion length inhibition). Collectively, this report will lead to a better understanding of the safety hazards found in soybean products in China and reveals the application of dipicolinic and kojic acids to reduce the use of carbendazim.

## 1. Introduction

In 2020, soybean production reached 353 million tons, with major producing areas in the USA, Brazil, Argentina and China [1]. Soybean-derived food products provide essential proteins, antioxidants and minerals, and are rich in isoflavones [2]. Several studies have shown that soy products can reduce serum concentrations of total cholesterol, while fermented soy products are effective in attenuating the effects of diabetes, blood pressure, cardiac disorders and cancer [3]. Despite their high nutritional and economic relevance, soybean plants are highly susceptible to a wide range of pathogens that reduce crop production and quality [4,5,6]. It has been estimated that, from 1996 to 2016, the total economic loss caused by soybean diseases in the USA was 95.48 billion dollars [7,8].

*Fusarium* species have been reported to attack different soybean tissues and cause a variety of diseases on soybean plants, such as wilting, root rot, seed rot and pod blight [9,10,11,12]. Some *Fusarium* species can infect different tissues. For example, *F. oxysporum* has been reported to cause soybean root rot, cotyledon spot and wilting [13,14,15]. Several screenings of soybean diseases have indicated that *Fusarium* is the most common fungal pathogen infecting soybean crops [16,17]. Sudden death syndrome, which is caused by *F. virguliforme*, is among the most important diseases affecting soybean production in the USA [18]. *F. acuminatum*, *F. avenaceum*, *F. commune*, *F. culmorum*, *F. equiseti*, *F. oxysporum*, *F. proliferatum*, *F. redolens*, *F. torulosum* and *F. tricinctum* were detected in diseased soybean roots in Alberta, Canada [19]. *F. avenaceum* was the most common species associated with soybean grains in Poland, followed by *F. equiseti* and *F. sporotrichioides* [20]. Recently, several *Fusarium* species, including *F. fujikuroi*, *F. luffae*, *F. proliferatum* and *F. sulawense*, were found associated with soybean pods in Hubei Province, China [21].

Apart from the enormous economic losses, some *Fusarium* strains can synthesize toxins, such as 4-acetyl nivalenol, 3-acetyl deoxynivalenol, diacetoxyscirpenol, neosolaniol, nivalenol and zearalenone [22]. Some of these toxins have been reported to induce anorexia, modify DNA by altering methylation and histone acetylation patterns and cause diabetes [23]. Fumonisins, which are synthesized by some *Fusarium* strains, cause fatal livestock diseases and are considered potentially carcinogenic mycotoxins for humans [24]. *F. sulawense*, among other species, has been reported to synthesize mycotoxin trichothecene, which can cause breathing problems and lung inflammation [25].

One of the main limitations in the control of soybean diseases caused by *Fusarium* species is the lack of information regarding the identity of the pathogens that are currently affecting soybean crops and the development of suitable methods for their management. In this study, *F. sulawense*, also named *F. sulawesiense*, was identified as the causal agent of soybean pod blight in Nantong municipality, China. Carbendazim, dipicolinic acid and kojic acid were evaluated as antifungal agents for *F. sulawense* management on soybean.

## 2. Materials and Methods

### 2.1. Sample Collection and Fungal Isolation

In November 2021, soybean pod blight symptoms were observed in Nantong municipality, China (120.54° E, 31.58° N). The symptoms were observed in nearly 69% of the pods from a commercial soybean field (3.2 ha). The field contained 3-month-old soybean plants in the harvest stage. Five small pieces of symptomatic soybean pods, approximately 0.3 cm^2^ in size, were extracted from different plants, surface sterilized in 1.5% NaOCl for 1 min and washed twice with sterile ddH_2_O. The pathogen was cultured on potato-dextrose-agar (PDA) medium containing chloramphenicol (50 µg/mL) in darkness at 26 °C for 5 days. PDA medium was prepared by boiling 200 g of potatoes in 1 L of water for 30 min. Then, the solution was filtered, and 20 g dextrose and 15 g agar were added (pH = 5.6). Three isolates, NJC31, NJC32 and NJC33 (NJC = Nantong, Jiangsu, China), were obtained via single spore isolation [26].

### 2.2. Morphological Characterization

A 5-mm-diameter mycelial PDA plug containing NJC31 was incubated in 50 mL yeast extract-peptone-dextrose (YEPD) medium (0.15 g yeast extract, 0.5 g peptone and 1 g glucose in 50 mL ddH_2_O) at 26 °C and 200 rpm in darkness for 48 h. Fungal morphology was observed using a Leica DM2500 microscope (Germany).

### 2.3. Sequence Analysis

The three isolates were grown in 100 mL potato-dextrose broth (PDB; prepared as PDA medium but in the absence of agar) at 26 °C and 200 rpm in darkness for 48 h. The genomic DNA was extracted using the Ezup Column Fungi Genomic DNA Purification Kit (Sangon Biotech, China) according to the manufacturer’s instructions. Internal transcribed spacer (*ITS*) region of rDNA, elongation factor 1-α (*EF1-α*) and RNA polymerase II second largest subunit (*RPB2*) partial sequences were amplified using the *ITS1/ITS4* (5′-TCCGTAGCTGAACCTGCGG-3′ and 5′-TCCTCCGCTTATTGATATGC-3′, respectively), *EF1-728F/EF1-986R* (5′-CATCGAGAAGTTCGAGAAGG-3′ and 5′-TACTTGAAGGAACCCTTACC-3′, respectively) and *fRPB2-7CF/fRPB2-11aR* (5′-ATGGGYAARCAAGCYATGGG-3′ and 5′-GCRTGGATCTTRTCRTCSACC-3′, respectively) primers [27,28,29].

Polymerase Chain Reaction (PCR) amplification was carried out in a 50 μL reaction mixture, which contained 25 μL of 2 × primestar, 1 μL of each primer (10 μM), 1 μL of DNA template (70 ng/μL) and 22 μL of ddH_2_O using a PCR Thermal Cycler (Techne HEMR 9600, China). The PCR conditions consisted of 2 min of initial denaturation at 98.0 °C, followed by 32 cycles of denaturation at 98 °C for 10 s; primer annealing 30 s at 53.8 °C for *ITS*, 56.8 °C for *EF1-α* and 55.9 °C for *RPB2*; extension at 72 °C for 80 s; and final extension at 72 °C for 2 min. Amplicons were checked by electrophoresis using a 1.0% agarose gel in TAE buffer (1×) and visualized with a UV luminometer (Tanon 1600R, China). DNA sequencing was performed by Sangon Biotech (Shanghai, China).

The obtained sequences were submitted to GenBank under accession numbers ON646105, ON646177 and ON646200 (*ITS*); ON685317-ON685319 (*EF1-α*); and ON685320-ON685322 (*RPB2*) (Table S1 and Figure S1).

### 2.4. Construction of the Phylogenetic Tree

The phylogenetic tree based on the *EF1-α* and *RPB2* sequences was constructed using MEGA7 with ex-type strains retrieved from GenBank. The *ITS* gene was not included in the phylogenetic tree due to the *ITS* gene of some ex-type isolates, including *F. lactis* CBS 411.97 and *F. parvisorum* CBS 137236, was not available in GenBank. After removing ambiguous regions and autapomorphic insertions manually, the alignment resulted in three matrices with a length of 594 (*EF1-α*) and 1646 (*RPB2*) sites. The evolutionary history was inferred using the Maximum Likelihood method based on the Tamura 3-parameter model. The percentage of trees in which the associated taxa clustered together is shown next to the branches. Initial trees for the heuristic search were obtained automatically by applying the Neighbor-Join and BioNJ algorithms to a matrix of pairwise distances estimated using the Maximum Composite Likelihood (MCL) approach, and then selecting the topology with a superior log likelihood value (−6447.23). A discrete Gamma distribution was used to model evolutionary rate differences among sites (5 categories (*+G*, parameter = 0.6742)). There were a total of 2241 positions in the final dataset. The number of bootstrap replications was 1000. The tree is drawn to scale, with branch lengths measured in the number of substitutions per site.

Apart from NJC31, NJC32 and NJC33, the *EF1-α* and *RPB2* sequences of 16 ex-type *Fusarium* isolates, including *F. sulawense* InaCC F964, *F. kotabaruense* InaCC F963, *F. tanahbumbuense* InaCC F965, *F. lacertarum* CBS 130185, *F. lactis* CBS 411.97, *F. mundagurra* NRRL 66235, *F. napiforme* CBS 748.97, *F. nygamai* CBS 749.97, *F. parvisorum* CBS 137236, *F. phyllophilum* CBS 216.76, *F. pseudocircinatum* CBS 449.97, *F. pseudonugamai* CBS 417.97, *F. ramigenum* CBS 418.98, *F. sudanense* CBS 454.97, *F. terricola* CBS 483.94 and *F. tjaetaba* NRRL 66243 [30], were used for the construction of the phylogenetic tree. *Lasiodiplodia theobromae* CBS 164.96 was used as an outgroup strain [31].

### 2.5. Pathogenicity Test

Re-inoculation of NJC31, NJC32 and NJC33 was carried out using detached ‘Lee68′ soybean pods. The pods were surface sterilized for 1 min in 1.5% NaOCl and washed twice with sterile ddH_2_O. A 0.5 cm^2^ size wound was cut in each soybean pod using a sterilized knife. The pathogens were inoculated using a 1 × 10^6^ spores/mL (20 μL) conidial suspension, which was prepared by extracting *F. sulawense* conidia from PDA medium. Each soybean pod contained one inoculation site. Sterilized ddH_2_O was used in the control experiment. Ten soybean pods were used for each isolate. Experiments were repeated 3 times. The pathogen was recovered from the wounds and the identity of the pathogen was confirmed via sequencing and morphology analyses, following Koch’s postulates.

The disease severity index (DSI) was calculated for each strain using a 3-point scale. The scores were 0 = no symptoms, 1 = mild symptoms (slight discoloration of pods), 2 = obvious lesions (pods were obviously diseased) and 3 = severe lesions (pods showing rot symptoms).

### 2.6. PCR Assay of Trichothecene Biosynthetic Genes

The trichothecene biosynthetic genes *TRI4*, *TRI5, TRI13NIV* and *TRI13DON* were amplified following the conditions reported by Stepien et al. [32,33]. The primers used in the amplification were 5′-CCCCTGGCTACTCTCGAGA-3′ and 5′-AAGCTTTGAGAACCTTCAC-3′ (*TRI4*), 5′-AGCGACTACAGGCTTCCCTC-3′ and 5′-AAACCATCCAGTTCTCCATCT-3′ (*TRI5*), 5′-CCAAATCCGAAAACCGCAG-3′ and 5′-TTGAAAGCTCCAATGTCGTG-3′ (*TRI13NIV*) and 5′-CATCATGAGACTTGTKRAGTTTGGG-3′ and 5′-GCTAGATCGATTGTTGCATTGAG-3′ (*TRI13DON*). The PCR amplification was carried out in a 50 μL reaction mixture, which contained 25 μL of 2 × primestar, 1 μL of each primer (10 μM), 1 μL of DNA template and 22 μL of ddH_2_O using a PCR Thermal Cycler (Techne HEMR 9600). The PCR conditions consisted of 15 min of initial denaturation at 94.0 °C, followed by 35 cycles of denaturation at 94 °C for 60 s, primer annealing at 58.0 °C for 30 s, extension at 72 °C for 60 s and final extension at 72 °C for 10 min.

PCR products were observed by electrophoresis using the procedure indicated in Section 2.3.

### 2.7. Efficacy of Carbendazim, Dipicolinic Acid and Kojic Acid for the Control of F. sulawense on Soybean Pods

Soybean pods were sterilized and wounded following the conditions indicated in Section 2.5. NJC31 was used in the screening. A 1 × 10^6^ NJC31 spores/mL (20 μL) conidial suspension was prepared as indicated in Section 2.5. For the curative assay, NJC31 conidia was poured into the wound, and the pods were maintained at 28 °C and 90% relative humidity for 24 h. Then, 10 mL of aqueous solutions containing carbendazim (3.8 mg/mL), dipicolinic acid (0.84 mg/mL) or kojic acid (0.71 mg/mL) were sprayed. Technical compounds (and not commercial fungicides) were used in the screening. The concentration of carbendazim was adjusted to the application rate recommended by suppliers, which suggests the use of 0.8 g carbendazim per kg of seeds for the management of *Fusarium* [34]. Dipicolinic (0.84 mg/mL) and kojic acids (0.71 mg/mL) were applied at the same concentrations previously reported [35,36]. After spraying the antifungal compounds, the soybean pods were kept at 28 °C and 90% relative humidity for 24 h. Sterilized ddH_2_O (10 mL) was sprayed in the control treatment. The efficacy of the fungicides was calculated according to the lesion length caused by NJC31. Twenty pods were used for each treatment condition. Experiments were repeated 3 times.

For the preventive assay, 10 mL aqueous solutions containing carbendazim (3.8 mg/mL), dipicolinic acid (0.84 mg/mL) or kojic acid (0.71 mg/mL) were sprayed on the wounded soybean pods. After drying at room temperature using a fan, a 1 × 10^6^ NJC31 spores/mL (20 μL) conidial suspension was injected into each wound. Sterilized ddH_2_O (10 mL) was sprayed in the control treatment. The soybean pods were incubated at 28 °C and 90% relative humidity for 72 h. Twenty pods were used per treatment. Experiments were repeated 3 times.

### 2.8. Combinations of Carbendazim, Dipicolinic Acid and Kojic Acid for the Control of F. sulawense on Soybean Pods

The following combinations were screened: (1) carbendazim (3.8 mg/mL)/dipicolinic acid (0.84 mg/mL); (2) carbendazim (1.9 mg/mL)/dipicolinic acid (0.84 mg/mL); (3) carbendazim (0.95 mg/mL)/dipicolinic acid (0.84 mg/mL); (4) carbendazim (3.8 mg/mL)/kojic acid (0.71 mg/mL); (5) carbendazim (1.9 mg/mL)/kojic acid (0.71 mg/mL); (6) carbendazim (0.95 mg/mL)/kojic acid (0.71 mg/mL); (7) dipicolinic acid (0.84 mg/mL)/kojic acid (0.71 mg/mL); (8) dipicolinic acid (0.21 mg/mL)/kojic acid (0.71 mg/mL); and (9) dipicolinic acid (0.84 mg/mL)/kojic acid (0.18 mg/mL). Their efficacy was screened in curative and preventive applications as described in Section 2.7. Sterilized ddH_2_O (10 mL) was sprayed in the control treatment. The soybean pods were incubated at 28 °C and 90% relative humidity for 72 h. Twenty pods were used per treatment condition. Experiments were repeated 3 times.

### 2.9. Combinations of Carbendazim, Dipicolinic Acid and Kojic Acid for the Control of F. sulawense on Soybean Pods

The protection provided by carbendazim and kojic acid for the control of NJC31 was evaluated at different time points. The preventive assay described in Section 2.7 was followed. The antifungals were applied at two different concentrations: (1) carbendazim (1.9 mg/mL)/kojic acid (0.71 mg/mL) and (2) carbendazim (0.95 mg/mL)/kojic acid (0.71 mg/mL). Then, the pathogen was inoculated after 0, 1, 2, 3, 4, 5, 6 and 7 days. The soybean pods were incubated at 28 °C and 90% relative humidity for 72 h after the inoculation of the pathogen. Twenty pods were used for each treatment condition. Experiments were repeated 3 times.

### 2.10. Statistical Analysis

Statistical analyses were performed using SPSS software v. 20.0 (IBM Corp., Armonk, NY, USA). The obtained data were processed using one-way ANOVA with post hoc multiple comparisons. Means were considered significantly different when *p* ≤ 0.05.

## 3. Results

### 3.1. F. sulawense Was Identified as the Causal Agent of Soybean Pod Blight in Nantong Municipality

**Figure 1:**
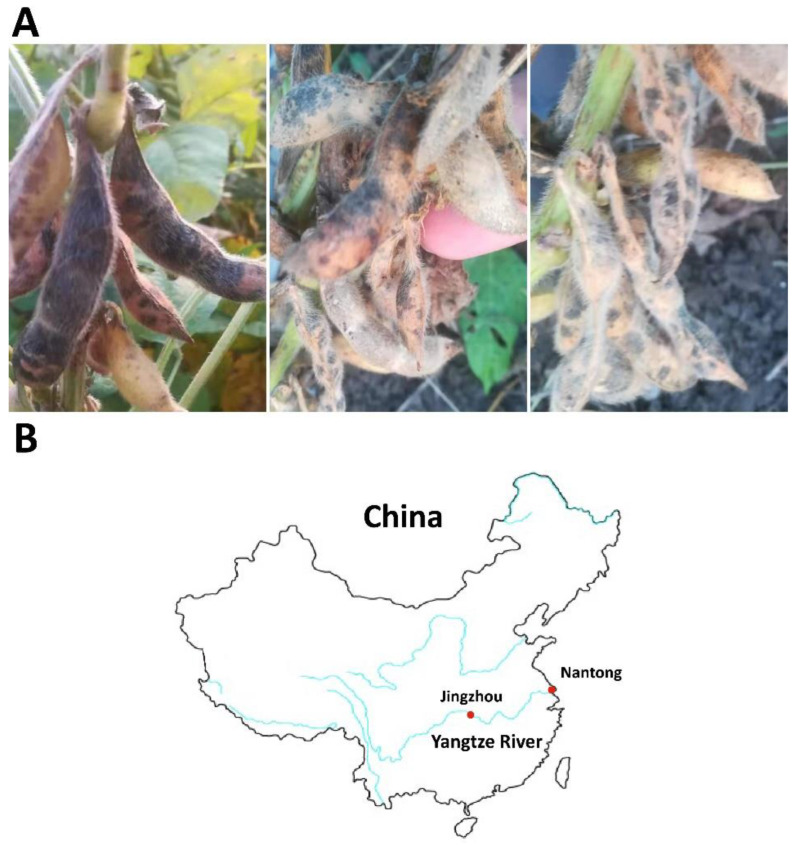
Symptoms caused by *Fusarium sulawense* and location. (**A**) Soybean pods (*Glycine max* cv. ‘Lee68’) in Nantong municipality exhibited black necrotic lesions and were heavily mildewed. (**B**) A map of China showing the municipalities of Nantong, Jiangsu Province and Jingzhou, Hubei Province. *F. sulawense* was identified as the causal agent of soybean pod blight in Nantong municipality in 2021 (this work) and in Jingzhou municipality in 2020 [21].

**Figure 2:**
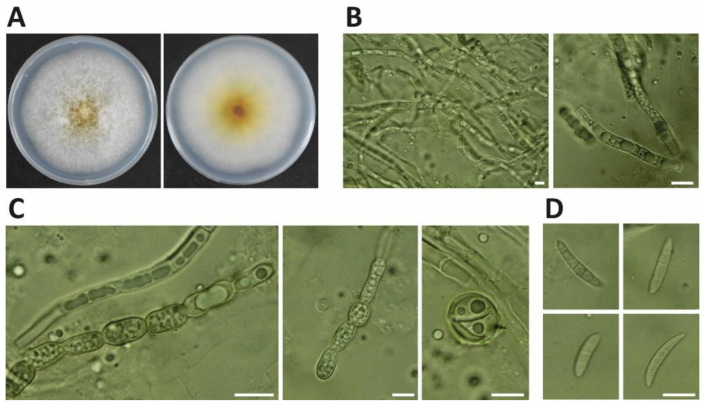
Morphological characteristics of *Fusarium sulawense* NJC31 isolated from soybean pods. (**A**) Colony of *F. sulawense* on PDA at 26 °C after incubation for 5 days. (**B**) Septate mycelium. (**C**) Linear and round chlamydospores (number of observations = 89). (**D**) Fusiform macroconidia (number of observations = 56) (10–15 μm in length) containing 3–5 septum separations. Bar = 10 μm.

In November 2021, soybean pods (*Glycine max* cv. ‘Lee68′) at the harvest stage exhibited black necrotic lesions (Figure 1). Three isolates, NJC31, NJC32 and NJC33, were obtained. The colonies of the three isolates showed white mycelium with yellow pigmentation on PDA (Figure 2). Microscope observations revealed the presence of septate mycelium. Linear (60–200 μm long) and round (approximately 10 μm diameter) chlamydospores were observed (number of observations = 89). Fusiform macroconidia, 10 to 15 µm in length, showed a curved shape and contained between 3 and 5 septum separations (number of observations = 56).

**Figure 3:**
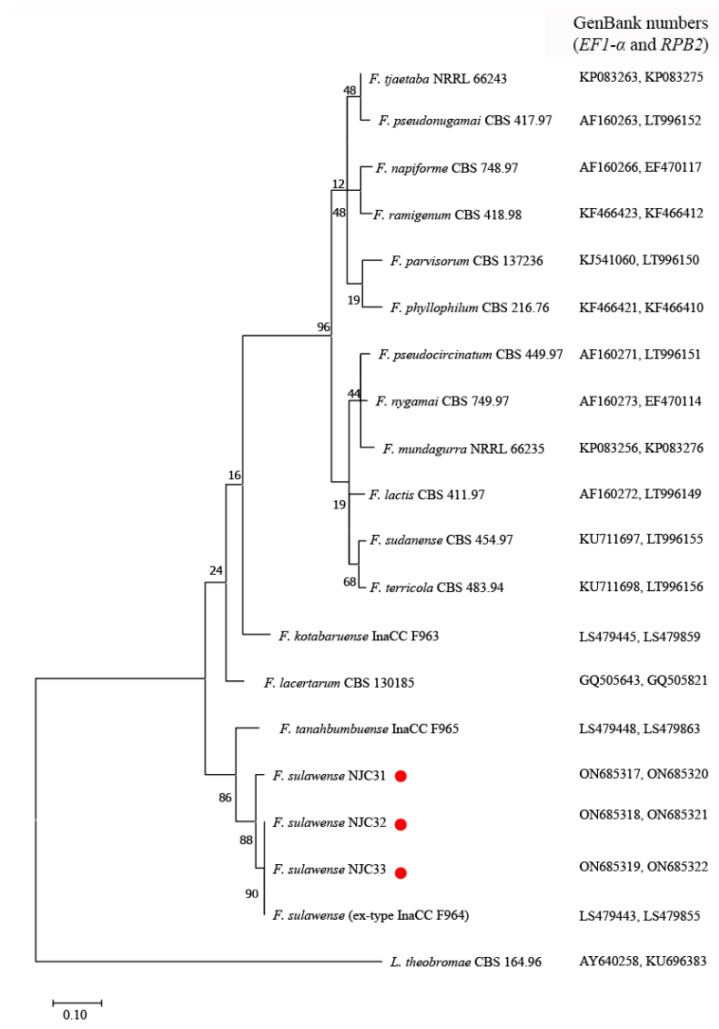
Phylogenetic analysis of elongation factor 1-α (*EF1-α*) and RNA polymerase II second largest subunit (*RPB2*) sequences from *Fusarium sulawense* strains NJC31, NJC32 and NJC33. The tree was constructed using MEGA7 with reference strains retrieved from GenBank. The evolutionary history was inferred using the Maximum Likelihood method and Tamura 3-parameter model. The tree with the highest log likelihood (−6447.23) is shown. A discrete Gamma distribution was used to model evolutionary rate differences among sites (5 categories (*+G*, parameter = 0.6742). The rate variation model allowed for some sites to be evolutionarily invariable ([+I], 20.81% sites). Default parameters were used in the analysis. The red points indicate the strains isolated in this work.

The *ITS* sequences of NJC31, NJC32 and NJC33 showed 100% identity compared to reference *F. sulawense* strains LC7919 and LC7920 (GenBank numbers: MK280811 and MK280805), which were also isolated in China [37]. The *EF1-α* sequence of NJC31 was 100% identical with *F. sulawense* LC7919, LC7920 and LC12149 (GenBank numbers: MK280811, MK280805 and MK280783), while the *EF1-α* sequences of NJC32 and NJC33 were 100% identical compared to the sequences from *F. sulawense* LC12176 and LC12169 (GenBank numbers: MK280839 and MK289756) [37]. The *RPB2* sequence of NJC31 showed 98.50% identity with respect to the *RPB2* sequences from *F. sulawense* LC7939, LC7919 and LC12176 (GenBank numbers: MK289795, MK289786 and MK289761); the *RPB2* sequence of NJC32 showed 99.89% homology with respect to the *RPB2* sequences from LC7939, LC7919 and LC12176; and the *RPB2* sequence of NJC33 showed 100% homology concerning the *RPB2* sequences from LC7939, LC7919 and LC12176 [37]. The amplified sequences showed 99.59–99.80% (*ITS*) and 97.63–100% (*EF1-α*) homology to the corresponding sequences of ex-type strain *F. sulawense* InaCC F964 (GenBank numbers: LS479410 and LS479443) [30]. Figure 3 shows a phylogenetic tree constructed by combining the *EF1-α* and *RPB2* sequences.

**Figure 4:**
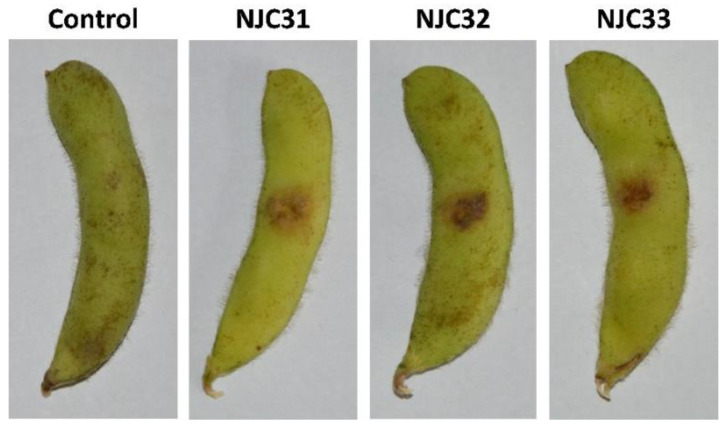
Pathogenicity assay of *Fusarium sulawense* strains NJC31, NJC32 and NJC33. A 0.5 cm^2^ size wound was cut in each soybean pod and the pathogens were inoculated using a 1 × 10^6^ spores/mL (20 μL) conidial suspension. Sterilized ddH_2_O was used in the control experiment. The pathogen was recovered from the wounds and the identity of the pathogen was confirmed via sequencing and morphology analyses, following Koch’s postulates.

A pathogenicity assay was carried out to confirm the virulence of the isolates (Figure 4). After inoculation of the strains, black-brown necrotic lesions were easily observed with the naked eye. The pathogen was recovered from the lesions and identified, confirming that *F. sulawense* was the causal agent of rot on soybean pods. The disease severity ranged from 75.1% to 88.4% for NJC31, 76.3% to 85.5% for NJC32 and 77.2% to 84.2% for NJC33, indicating that the three isolates were highly virulent.

**Figure 5:**
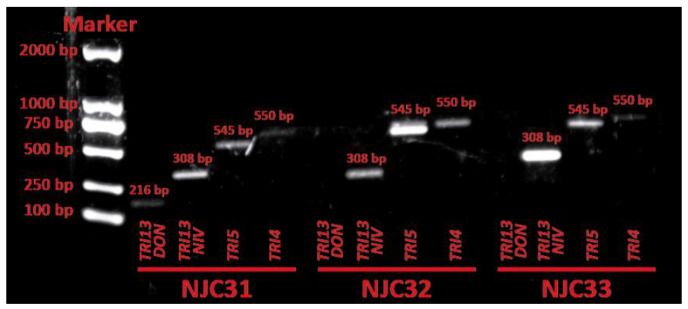
Electrophoresis analysis of trichothecene biosynthetic genes *TRI13DON*, *TRI13NIV*, *TRI5* and *TRI4* in *Fusarium sulawense* strains NJC31, NJC32 and NJC33. The product sizes predicted for *Fusarium equiseti* were used to identify tentatively the PCR products [32].

The ability of NJC31, NJC32 and NJC33 to synthesize trichothecenes was evaluated by amplifying the trichothecene biosynthesis genes *TRI4*, *TRI5*, *TRI13NIV* and *TRI13DON*. The presence of the *TRI4* and *TRI5* genes is specific for A-trichothecene producers, while the *TRI13NIV* and *TRI13DON* genes are involved in the biosynthesis of B-type trichothecenes nivalenol and deoxynivalenol, respectively [32,33]. The product sizes predicted for *F. equiseti* were used to identify tentatively the PCR products from NJC31, NJC32 and NJC33 [32]. The PCR assay showed the presence of *TRI4*, *TRI5*, *TRI13NIV* and *TRI13DON* in the DNA of NJC31, while *TRI4*, *TRI5* and *TRI13NIV* were detected in NJC32 and NJC33 (Figure 5). These results suggest that NJC31 can synthesize A-type trichothecenes, nivalenol and deoxynivalenol, while NJC32 and NJC33 seem to produce only A-type trichothecenes and nivalenol. Unfortunately, no trichothecene biosynthesis genes were obtained in sufficient concentration to allow for sequencing.

### 3.2. Combinations of Carbendazim, Dipicolinic Acid and Kojic Acid Reduced the Symptoms of F. sulawense on Soybean Pods

The preventive and curative efficacies of carbendazim, dipicolinic acid and kojic acid were evaluated by applying the compounds separately and in combination. For the preventive assay, the soybean pods were treated with the fungicides, and then *F. sulawense* was inoculated. For the curative assay, *F. sulawense* was inoculated on the soybean pods. After the appearance of the disease symptoms, the antifungal compounds were applied. The curative and preventive assays were performed using NJC31.

**Figure 6:**
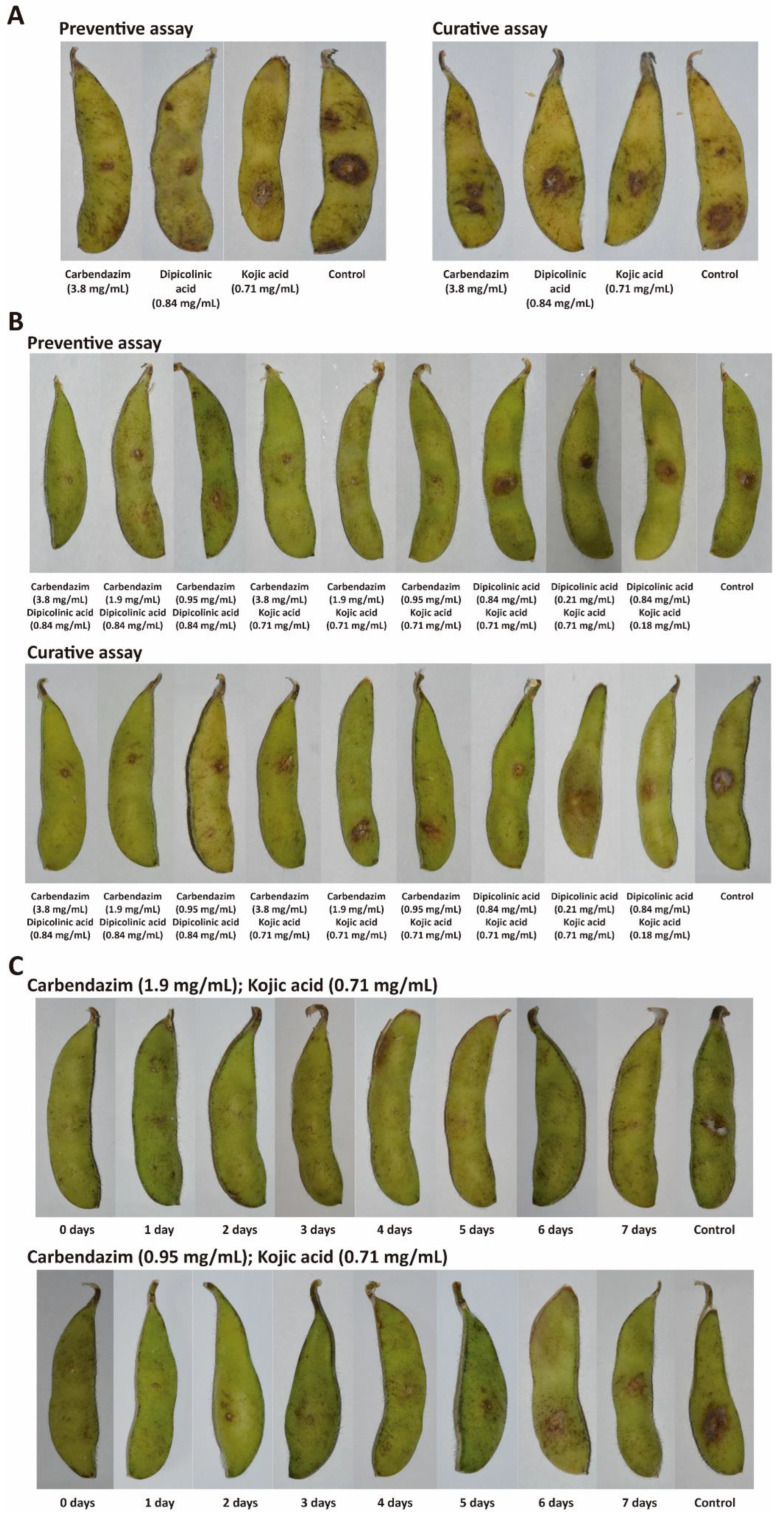
Images showing the efficacy of carbendazim, dipicolinic acid and kojic acid for the control of *Fusarium sulawense* NJC31 symptoms on soybean pods. (**A**) Symptoms after treatment with carbendazim, dipicolinic acid and kojic acid separately. (**B**) Symptoms after applying combinations of carbendazim, dipicolinic acid and kojic acid. (**C**) Symptoms after preventive application with carbendazim and kojic acid. The pathogen was inoculated at different time points after treatment with the fungicides. Control experiments were carried out in the absence of fungicides.

Although carbendazim, dipicolinic acid and kojic acid showed preventive efficacy when they were applied separately, none of the fungicides showed curative efficacy. The highest preventive efficacy was observed when using carbendazim (76.2% lesion length inhibition) (Table 1 and Figure 6). In contrast, dipicolinic acid (33.3% lesion length inhibition) and kojic acid (28.6% lesion length inhibition) showed weak preventive efficacies.

To continue the screening, carbendazim was combined with dipicolinic and kojic acids (Table 2 and Figure 6). Although none of the fungicides showed curative efficacy when they were applied separately, the combinations of carbendazim and dipicolinic acid reduced the lesion length caused by *F. sulawense* (although the means were not significantly different). In contrast, the combinations of carbendazim and kojic acid were not effective for controlling the pathogen, while the application of 0.84 mg/mL dipicolinic acid and 0.71 mg/mL kojic acid reduced the lesion length by 33.3%.

In preventive applications, the combination of 3.8 mg/mL carbendazim and 0.84 mg/mL dipicolinic acid (82.7% lesion length inhibition) slightly increased the efficacy when compared to the single application of carbendazim (76.2% lesion length inhibition). The highest efficacy was detected when using 1.9 mg/mL carbendazim and 0.71 mg/mL kojic acid (84.9% lesion length inhibition). The concentration of carbendazim could be reduced by combining this fungicide with dipicolinic and kojic acids without an obvious decrease in antifungal activity.

Combinations of 1.9 mg/mL carbendazim and 0.71 mg/mL kojic acid could protect soybean pods from *F. sulawense* infection for 7 days without any obvious decrease in efficacy (Table 3 and Figure 6). The inhibitory activity ranged from 76.2% to 81.7% during the 7 days of study. These results indicate that the combination of 1.9 mg/mL carbendazim and 0.71 mg/mL kojic acid can be a suitable mixture to provide extended protection of soybean pods. In contrast, the combination of 0.95 mg/mL carbendazim and 0.71 mg/mL kojic acid provided limited protection, with only 50.8% and 26.9% inhibitory activities after 4 and 6 days, respectively.

## 4. Discussion

*F. sulawense* is a novel *Fusarium* species that was designated in 2019 [30,38]. *F. sulawense* was isolated for the first time on bananas in Indonesia in 2014, and it has been indicated that this species is endemic to this crop [30]. This species belongs to the *F. incarnatum-equiseti* complex, which is a phylogenetically rich complex that contains more than 30 pathogenic species [39]. *F. sulawense* has been reported to infect numerous crops in tropical and equatorial areas. For example, it was reported to cause rot on melon fruits in Brazil in 2020 [40]. *F. sulawense* was also found to cause rot on papaya, leaf spot on mango and blight on plums in China [41,42,43]. This species was identified as the causal agent of malformations on *Swietenia macrophylla* and head blight of wheat in Mexico [25,27]. *F. sulawense* was discovered to be the cause of sesame stem rot in Pakistan [44], and rice sheath rot in Indonesia [45]. As indicated in the Introduction Section, *F. sulawense* was reported to cause soybean pod blight in Hubei, China, in July–August 2020 [30]. In agreement with the symptoms observed in Hubei, *F. sulawense* caused black-brown necrotic lesions on soybean pods in Nantong municipality, and the pods were heavily mildewed. Hubei and Jiangsu are located in subtropical areas on the Yangtze River, confirming the spread of *F. sulawense* to higher latitudes. This study suggests that *F. sulawense* may become an important soybean pathogen across China in the coming years. All spreads caused by *F. sulawense* in China, Brazil and Mexico were reported from 2020 to date, suggesting that this is a new pathogen in these countries and has spread from Indonesia to other parts of the world. According to the reports, this species can only be found in certain plants but does not have a cosmopolitan distribution. *F. graminearum*, *F. pseudograminearum* and *F. meridionale* have also been identified as the causal agents of soybean pod blight in Argentina and Canada [12,46]. Some *Diaporthe* species, such as *D. aspalathi*, *D. sojae* and *D. longicolla*, have been reported to be among the main pathogens producing soybean pod blight in Canada and Brazil [47,48,49].

Morphology analyses confirmed the presence of microconidia, fusiform macroconidia, septate mycelium and chlamydospores, which are consistent with the common morphology of *Fusarium* species [29,50,51]. Maryani et al. analyzed the morphological characteristics of the *F. sulawense* ex-type strain InaCC F964 [30]. This strain also formed white mycelium with yellow pigmentation on PDA medium, and showed very similar structures, such as 10–15 μm conidia and elongated chlamydospores. The number of septum separations in the conidia was similar, 3-6 septum separations, and in both cases the conidia showed a curved shape. Although the morphology analysis indicated that the strains isolated in this study showed very similar morphology to *F. sulawense* ex-type InaCC F964, this analysis could not confirm the specific species given that most *Fusarium* species show similar morphological characteristics. For this reason, a sequencing analysis was carried out. This analysis was performed by sequencing the *ITS*, *EF1-α* and *RPB2* genes, which are common genes used for the identification of *Fusarium* species [52,53].

Zhao et al. indicated that the identified strains from the *F. fujikuroi* species complex showed higher virulence on soybean pods than the strains from the *F. incarnatum-equiseti* species complex [21]. In that study, the disease incidence ranged from 63.3% to 80%, which is similar to the disease incidence observed in this study (69%). Although *F. proliferatum* 39 YZU 201408 was reported to exhibit high virulence with 72.2–90.0% disease severity, the virulence of the *F. sulawense* strains isolated in Hubei was under 50% [21]. The *F. sulawense* strains isolated in this work showed higher virulence (75.1–88.4% disease severity) compared to the *F. sulawense* strains isolated in Hubei; however, both assays were not carried out using the same soybean variety. Further research is necessary to examine and compare the virulence of different *Fusarium* species on soybean pods. In this report, the three isolates were *F. sulawense* strains, suggesting that *F. sulawense* is the main agent causing soybean pod blight symptoms in Nantong. However, it would be interesting to extend the screening to more fields and plants in the area to identify all the pathogens and evaluate the spread of this disease in Jiangsu Province.

*F. incarnatum-equiseti* species are known to synthesize mycotoxins, such as zearalenone and trichothecene [22,25,54]. Although isolates NJC31, NJC32 and NJC33 were found to contain several trichothecene biosynthesis genes, further chemical analyses are necessary to confirm the presence of trichothecenes in the secretions of and their structures. The ability of *F. sulawense* to produce trichothecenes was previously reported by Montoya-Martinez et al. [25]. As far as we know, *F. sulawense* has never been reported to synthesize zearalenone. However, further studies are necessary to confirm this chemotype. Trichothecenes are also synthetized by *F. graminearum* and are commonly found in cereal grains [55]. Although A-type trichothecenes are more toxic to mammals than B-type trichothecenes, B-type trichothecenes are more common [56]. Trichothecenes can produce hypoxia, which in turn promotes oxidative stress in human cells [57]. The toxic effects of trichothecenes are stronger in growing children than in adults [58]. The obtained results suggest that the *F. sulawense* strains isolated in Nantong can produce both A-type and B-type trichothecenes, indicating that the presence of these *F. sulawense* isolates on soybean pods may pose important health hazards. For this reason, the occurrence of *F. sulawense* on soybean must be carefully monitored and controlled.

Carbendazim, which shows a mode of action based on the inhibition of microtubule polymerization in fungal cells [59], is a commercial fungicide commonly used for the management of soybean diseases [60]. Despite carbendazim’s broad range of antifungal activity and wide use, the production and use of carbendazim have been restricted in several countries due to its toxic effects [61]. Carbendazim has been related to major human diseases, such as cancer [62], and is known to produce embryotoxicity, germ cell apoptosis, teratogenesis, infertility, hepatocellular dysfunction, hematopoiesis and developmental toxicity in different mammalian species [63]. For these reasons, methods to decrease the use of this fungicide are urgently needed. On the other hand, dipicolinic and kojic acids are experimental compounds and their antifungal activities have been screened against different fungal plant pathogens [35,36,64]. As far as we know, no toxic effects were reported for these compounds, which is an important advantage compared to carbendazim. Dipicolinic and kojic acids are unexpensive (dipicolinic acid ≈ 1.3 EUR/g; koijc acid ≈ 0.3 EUR/g), showing similar price in comparison to carbendazim (≈ 0.9 EUR/g), and are produced in large scale. Kojic acid has been reported to show higher stability than dipicolinic acid and carbendazim in pear fruit [64]. In contrast with carbendazim, which is produced by chemical synthesis, dipicolinic and kojic acids are natural secondary metabolites. Dipicolinic acid is produced by *Bacillus* species and is a common component of bacterial spores [65], while kojic acid is synthesized by some *Aspergillus* strains and is widely used in the cosmetic industry to protect skin from UV radiation [66]. Dipiconilic acid was reported to inhibit chitin synthesis in *Valsa pyri* [35], whereas kojic acid was demonstrated to block the biosynthesis of melanin in *Sclerotinia sclerotiorum* [36].

All antifungal compounds showed higher preventive than curative efficacies. Separately, none of the compounds could reduce the disease symptoms in the curative assay. This highlights the difficulty in controlling *F. sulawense* after observing rot symptoms. Although none of the compounds showed any curative efficacy against the pathogen, the combination of dipicolinic acid and carbendazim reduced soybean pod blight symptoms. Interestingly, the combination of dipicolinic acid and carbendazim enhanced the curative and preventive efficacies of the commercial fungicide. These results suggest that dipicolinic acid can be a suitable complement to reduce the concentration and use of carbendazim. These results can be explained considering that dipicolinic acid is capable of damaging *F. sulawense* cell wall, which may facilitate the entry of carbendazim inside the pathogen cells [67]. As far as we know, this is the first study regarding the combination of carbendazim and dipicolinic acid for the control of fungal diseases. Previously, it was observed that combining carbendazim and kojic acid enhanced the ability of the commercial fungicide to control *S. sclerotiorum* on soybean pods [36].

Interestingly, combinations of carbendazim and kojic acid showed high protection ability inhibiting the pathogen’s advancement for 7 days. This highlights the potential application of this combination to control *F. sulawense* on soybean pods. It must be noted that the maturation of soybean pods requires approximately three weeks, and during this time, the pods are highly susceptible to fungal pathogen infections [68,69].

As far as we know, this is the first method for the management of *F. sulawense*. Only one study regarding the management of *Fusarium* species on soybean was reported to date and involved the use of captan, fludioxonil, azoxystrobin, trifloxystrobin and pyraclostrobin for the control of *F. graminearum* on soybean [70]. The highest efficacies were observed when using captan and fludioxonil. In contrast with the previous report, this study also involved the use of natural compounds.

## 5. Conclusions

In summary, new *F. sulawense* strains were identified in Nantong municipality as the causal agents of soybean pod rot. Given that, in 2020, *F. sulawense* was also found causing rot on soybean pods cultivated in Hubei, this new report suggests that *F. sulawense* may become a common soybean pathogen in China over the next few years. Several biosynthesis genes were amplified in the *F. sulawense* isolates, suggesting that they may be able to produce A-type and B-type trichothecenes and, for this reason, can pose an important safety hazard. Carbendazim, dipicolinic acid and kojic acid were screened for the control of *F. sulawense* on soybean, revealing that combinations of carbendazim and dipicolinic acid, and carbendazim and kojic acid, are a suitable method to control this pathogen. Although combinations of carbendazim and kojic acid only showed preventive efficacy, combinations of carbendazim and dipicolinic acid showed both preventive and curative abilities. This is the first study regarding the combination of carbendazim and dipicolinic acid. The obtained results suggest that dipicolinic and kojic acids can be suitable complements to reduce the use of carbendazim. Collectively, this report will lead to a better understanding of the potential safety hazards found in soybean products in China and reveals the potential use of natural metabolites dipicolinic and kojic acids for the control of *Fusarium* species in soybean.

## Figures and Tables

**Table 1:** Efficacy of carbendazim, dipicolinic acid and kojic acid against *Fusarium sulawense* on soybean pods.

Application	Antifungal Agent	Concentration (mg/mL)	Lesion Length (mm) ^1,2^	Inhibitory Activity (%)
Preventive	Carbendazim	3.80	0.95 ± 0.38 b	76.2
	Dipicolinic acid	0.84	2.78 ± 1.20 ab	33.3
	Kojic acid	0.71	3.02 ± 1.67 ab	28.6
	Control ^3^	-	4.27 ± 1.93 a	-
Curative	Carbendazim	3.80	4.17 ± 1.93 a	-
	Dipicolinic acid	0.84	4.77 ± 2.16 a	-
	Kojic acid	0.71	4.63 ± 2.25 a	-
	Control ^3^	-	4.00 ± 1.91 a	-

^1^ Lesion length (n = 60) ± SD. ^2^ Differences between means were considered significant when *p* ≤ 0.05. Curative and preventive assays were considered different groups. ^3^ Control experiment was carried out in the absence of fungicides.

**Table 2:** Efficacy of carbendazim, dipicolinic acid and kojic acid combinations against *Fusarium sulawense* on soybean pods.

Application	Antifungal Agent (Concentration; mg/mL)	Lesion Length (mm) ^1,2^	Inhibitory Activity (%)
Preventive	Carbendazim (3.8); Dipicolinic acid (0.84)	0.77 ± 0.49 b	82.7
	Carbendazim (1.9); Dipicolinic acid (0.84)	1.25 ± 0.60 b	71.8
	Carbendazim (0.95); Dipicolinic acid (0.84)	1.30 ± 0.72 b	70.7
	Carbendazim (3.8); Kojic acid (0.71)	0.80 ± 0.25 b	82.0
	Carbendazim (1.9); Kojic acid (0.71)	0.67 ± 0.48 b	84.9
	Carbendazim (0.95); Kojic acid (0.71)	4.17 ± 1.97 a	6.0
	Dipicolinic acid (0.84); Kojic acid (0.71)	3.57 ± 1.29 a	19.5
	Dipicolinic acid (0.21); Kojic acid (0.71)	4.30 ± 1.74 a	3.0
	Dipicolinic acid (0.84); Kojic acid (0.18)	4.11 ± 1.51 a	7.1
	Control ^3^	4.43 ± 1.52 a	-
Curative	Carbendazim (3.8); Dipicolinic acid (0.84)	2.41 ± 1.47 a	49.1
	Carbendazim (1.9); Dipicolinic acid (0.84)	2.62 ± 1.25 a	44.7
	Carbendazim (0.95); Dipicolinic acid (0.84)	2.68 ± 1.88 a	43.3
	Carbendazim (3.8); Kojic acid (0.71)	3.48 ± 1.62 a	26.4
	Carbendazim (1.9); Kojic acid (0.71)	4.83 ± 2.33 a	-
	Carbendazim (0.95); Kojic acid (0.71)	4.40 ± 1.84 a	7.0
	Dipicolinic acid (0.84); Kojic acid (0.71)	3.17 ± 1.68 a	33.1
	Dipicolinic acid (0.21); Kojic acid (0.71)	4.38 ± 2.49 a	7.4
	Dipicolinic acid (0.84); Kojic acid (0.18)	4.57 ± 2.61 a	3.5
	Control ^3^	4.73 ± 1.44 a	-

^1^ Lesion length (n = 60) ± SD. ^2^ Differences between means were considered significant when *p* ≤ 0.05. Curative and preventive assays were considered different groups. ^3^ Control experiment was carried out in the absence of fungicides.

**Table 3:** Preventive efficacy of carbendazim and kojic acid combinations for the control of *Fusarium sulawense* on soybean pods at different time points.

Antifungal Agent (Concentration; mg/mL)	Time between Application of the Fungicides and Inoculation of the Pathogen (days)	Lesion length (mm) ^1,2^	Inhibitory Activity (%)
Carbendazim (1.9 mg/mL); Kojic acid (0.71 mg/mL)	0	0.93 ± 0.70 b	77.6
	1	0.99 ± 0.74 b	76.2
	2	0.90 ± 0.55 b	78.4
	3	0.78 ± 0.31 b	81.3
	4	0.76 ± 0.49 b	81.7
	5	0.94 ± 0.45 b	77.5
	6	0.86 ± 0.45 b	79.4
	7	1.11 ± 0.64 b	73.3
	Control ^3^	4.16 ± 0.98 a	-
Carbendazim (0.95 mg/mL); Kojic acid (0.71 mg/mL)	0	1.02 ± 0.90 c	77.5
	1	0.73 ± 0.69 c	83.9
	2	1.77 ± 0.73 bc	60.9
	3	2.27 ± 0.99 bc	49.9
	4	2.23 ± 1.19 bc	50.8
	5	2.52 ± 1.21 abc	44.4
	6	3.31 ± 1.27 ab	26.9
	7	3.40 ± 1.37 ab	24.9
	Control ^3^	4.53 ± 1.12 a	-

^1^ Lesion length (n = 60) ± SD. ^2^ Differences between means were considered significant when *p* ≤ 0.05. Curative and preventive assays were considered different groups. ^3^ Control experiment was carried out in the absence of fungicides.

## Data Availability

Not applicable.

## Supplementary Materials

The following supporting information can be downloaded at: https://www.mdpi.com/article/10.3390/ijerph191710531/s1, Table S1: Sequences of the *Fusarium sulawense* strains isolated from soybean pods in China, Figure S1: Agarose gel showing the amplicons of *ITS*, *EF1-α*, *RPB2* genes.
