# Supplementary material for: Identification of New Fusarium sulawense Strains Causing Soybean Pod Blight in China and Their Control Using Carbendazim, Dipicolinic Acid and Kojic Acid
Source: Int J Environ Res Public Health. 2022 Aug 24;19(17):10531. doi: 10.3390/ijerph191710531 (PMC9518069; doi:10.3390/ijerph191710531)
Supplement: Supplementary file 1 [file ijerph-19-10531-s001.zip › ijerph-1844219-supplementary.pdf]

Figure S1

Agarose gel showing the amplicons of *ITS*, *EF1-α* and *RPB2* genes. The amplification conditions are detailed in the manuscript in Section 2.3.

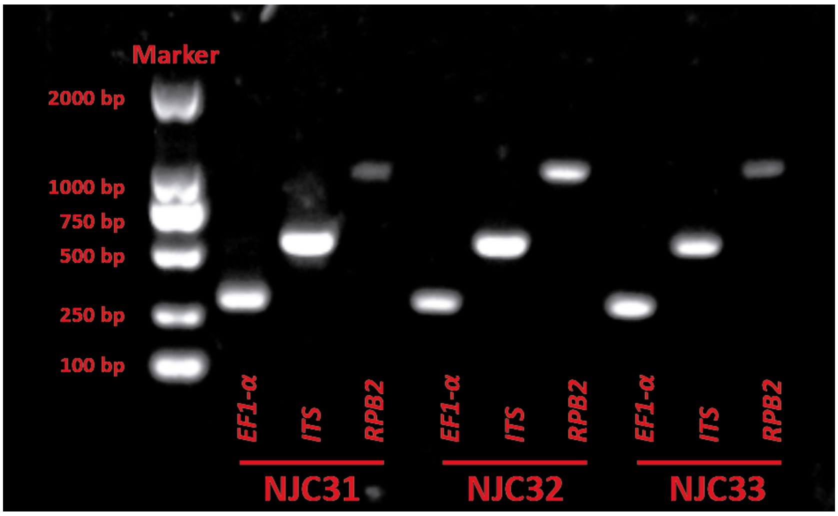

Table S1

Sequences of the *Fusarium sulawense* strains isolated from soybean pods in China

***ITS* genes:**

>ITS *Fusarium sulawense* NJC31

ACCCCTGTGACATACCTATACGTTGCCTCGGCGGATCAGCCCGCGCCCCGTAAAACGGGA
CGGCCCGCCCGAGGACCCCTAAACTCTGTTTTTAGTGGAACTTCTGAGTAAAACAAACAA
ATAAATCAAAACTTTCAACAACGGATCTCTTGGTTCTGGCATCGATGAAGAACGCAGCAA
AATGCGATAAGTAATGTGAATTGCAGAATTCAGTGAATCATCGAATCTTTGAACGCACAT
TGCGCCCGCCAGTATTCTGGCGGGCATGCCTGTTCGAGCGTCATTTCAACCCTCAAGCTC
AGCTTGGTGTTGGGACTCGCGGTAACCCGCGTTCCCCAAATCGATTGGCGGTCACGTCGA
GCTTCCATAGCGTAGTAATCATACACCTCGTTACTGGTAATCGTCGCGGCCACGCCGTTA
AACCCCAACTTCTGAATGTTGACCTCGGATCAGGTAGGAATACCCGCTGAACTTAAGCAT
ATCAATAA

>ITS *Fusarium sulawense* NJC32

CCTGTGACATACCTATACGTTGCCTCGGCGGATCAGCCCGCGCCCCGTAAAACGGGACGG
CCCGCCCGAGGACCCCTAAACTCTGTTTTTAGTGGAACTTCTGAGTAAAACAAACAAATA
AATCAAAACTTTCAACAACGGATCTCTTGGTTCTGGCATCGATGAAGAACGCAGCAAAAT
GCGATAAGTAATGTGAATTGCAGAATTCAGTGAATCATCGAATCTTTGAACGCACATTGC
GCCCGCCAGTATTCTGGCGGGCATGCCTGTTCGAGCGTCATTTCAACCCTCAAGCTCAGC
TTGGTGTTGGGACTCGCGGTAACCCGCGTTCCCCAAATCGATTGGCGGTCACGTCGAGCT
TCCATAGCGTAGTAATCATACACCTCGTTACTGGTAATCGTCGCGGCCACGCCGTTAAAC
CCCAACTTCTGAATGTTGACCTCGGATCAGGTAGGAATACCCGCTGAACTTAAGCATATC
AATA

>ITS *Fusarium sulawense* NJC33

TCCCAACCCCTGTGACATACCTATACGTTGCCTCGGCGGATCAGCCCGCGCCCCGTAAAA
CGGGACGGCCCGCCCGAGGACCCCTAAACTCTGTTTTTAGTGGAACTTCTGAGTAAAACA
AACAAATAAATCAAAACTTTCAACAACGGATCTCTTGGTTCTGGCATCGATGAAGAACGC
AGCAAAATGCGATAAGTAATGTGAATTGCAGAATTCAGTGAATCATCGAATCTTTGAACG
CACATTGCGCCCGCCAGTATTCTGGCGGGCATGCCTGTTCGAGCGTCATTTCAACCCTCA
AGCTCAGCTTGGTGTTGGGACTCGCGGTAACCCGCGTTCCCCAAATCGATTGGCGGTCAC
GTCGAGCTTCCATAGCGTAGTAATCATACACCTCGTTACTGGTAATCGTCGCGGCCACGC
CGTTAAACCCCAACTTCTGAATGTTGACCTCGGATCAGGTAGGAATACCCGCTGAACTTA
AGCATA

***EF1-α* genes:**

>EF1-a *Fusarium sulawense* NJC31

CATCGAGAAGTTCGAGAAGGTTGGTTTCCATTTCCCCGATCGCACGCCCTCTACCCACCG
ATCCATCAGTCGAATCAGTTACGACGATTGAATATGCGCCTGTTACCCCGCTCGAGTACA
AAATTTTGCGGTTCAACCGTAATTTTTTTTGGTGGGGTTTCAACCCCGCCACTCGAGCGA
CAGACGTTTGCCCTCTTCCCAGAAACCCATGTCTTGTGCATCACGTGTCCATCAGCCACT
AACCACCCGACAAAGAGC

>EF1-a *Fusarium sulawense* NJC32

TCATCGAGAAGTTCGAGAAGGTTGGTTTCCATTTCCCCGATCGCACGCCGTCTACCCACC
GATCCATCAGTCGAATCAGTTACGACGATTGAATATGCGCCTGTTACCCCGCTCGAGTAC
AAAATTTTGCGGTTCAACCGTAATTTTTTTGGTGGGGTTTCAACCCCGCTACTCGAGCGA
CAGACGTTTGCCCTCTTCCCACAAACTCATGTCTCGTGCATCACGTGTCCATCAGCCACT
AACCACCCGACA

>EF1-a *Fusarium sulawense* NJC33

TCATCGAGAAGTTCGAGAAGGTTGGTTTCCATTTCCCCGATCGCACGCCGTCTACCCACC
GATCCATCAGTCGAATCAGTTACGACGATTGAATATGCGCCTGTTACCCCGCTCGAGTAC
AAAATTTTGCGGTTCAACCGTAATTTTTTTGGTGGGGTTTCAACCCCGCTACTCGAGCGA
CAGACGTTTGCCCTCTTCCCACAAACTCATGTCTCGTGCATCACGTGTCCATCAGCCACT
AACCACCCGACA

<u>*RPB2* genes:</u>

>RPB2 *Fusarium sulawense* NJC31

CGACGTATGGATACTATGGCCAATATTCTCTACTATCCGCAAAAGCCTCTCGCCACCACC
CGATCCATGGAGTTCCTCAAGTTTCGTGAATTGCCTGCCGGTCAGAACGCCATTGTTGCT
ATCGCTTGTTATTCAGGATACAACCAGGAAGATTCCGTCATTATGAACCAGAGCAGTATT
GATCGAGGCCTGTTCCGCAGTCTCTTCTTCCGATCATACTCGGATCAGGAGAAGAAGGTC
GGTCTAAACTACACAGAAATCTTCGAGAAACCCTTCCAACAAACAACACTGCGAATGAAG
CATGGAACATACGACAAGCTCGACGAGGATGGTATTGTGGCTCCTGGTGTGCGAGTGTCA
GGTGAAGATATCATTATTGGCAAGACTGCGCCTATCGACCAGGAGAATCAAGATCTCGGT
ACCAGAACTCAGTCGCACCAGCGCCGCGATATCTCTACACCTCTGCGAAGTACAGAGAAC
GGTATTGTTGATCAAGTCATCTTGACCGTCAACGCCGACAATGTCAAATACGTCAAGGTC
CGAGTACGAACAACCAAGATCCCCCAAATTGGTGACAAGTTTGCTTCTCGTCACGGTCAA
AAGGGTACAATCGGTGTAACTTACCGACAGGAGGATATGCCCTTCAGCAGAGAGGGTCTG
ACTCCCGATATTATTATCAACCCTCACGCCATTCCATCTCGAATGACAATTGCCCATTTG
ATTGAGTGTCTGCTAAGTAAAGTCTCAACACTTGAGGGTATGGAGGGTGACGCGACGCCC
TTCACCGATGTCACTGTCGATTCCGTGTCGGAACTTCTGAGAAAGCACGGCTACCAGTCT
CGAGGTTTCGAGGTTATGTACAATGGTCACACTGGACGTAAGCTCCGCGCCCAGGTCTTC
TTTGGACCTACATACTACCAACGACTTCGTCACATGGT

>RPB2 *Fusarium sulawense* NJC32

AGCCACCACCAGATCCATGGAGTTCCTCAAGTTCCGTGAATTGCCTGCTGGTCAGAACGC
CATTGTTGCTATCGCTTGTTATTCAGGATACAACCAGGAAGATTCCGTCATTATGAACCA
GAGCAGTATTGACCGAGGCCTGTTCCGCAGTCTCTTCTTCCGATCATACTCGGATCAGGA
GAAGAAGGTCGGTCTAAACTACACAGAAATCTTCGAGAAGCCCTTCCAACAAACAACGCT
TCGAATGAAGCATGGAACATACGACAAGCTCGACGAGGATGGTATTGTGGCTCCTGGTGT
GCGAGTGTCAGGTGAAGATATCATCATTGGCAAGACTGCGCCTATCGACCAAGAGAATCA
AGATCTCGGTACCAGAACTCAGTCGCACCAGCGCCGCGATATCTCTACACCTCTGCGAAG
TACAGAGAACGGTATTGTTGATCAAGTCATTTTGACCGTCAACGCCGACAATGTCAAATA
CGTCAAGGTCCGAGTACGAACAACCAAGATCCCCCAGATTGGTGACAAGTTTGCTTCTCG

TCACGGTCAAAAGGGTACAATCGGTGTAACTTACCGACAGGAGGATATGCCCTTCAGCAG
AGAGGGTCTGACTCCCGATATTATTATCAACCCTCACGCCATTCCATCTCGAATGACAAT
TGCCCATTTGATTGAGTGTCTGCTAAGTAAAGTCTCAACACTTGAGGGTATGGAGGGTGA
CGCGACGCCCTTTACCGATGTCACTGTCGATTCCGTGTCGGAACTTCTGAGAAAGCACGG
CTACCAGTCTCGAGGTTTCGAGGTTATGTACAATGGTCACACTGGACGTAAGCTCCGCGC
CCAAGTCTTCTTTGGACCTACATACTACCAGCGACTTCGTCACATGGGTCGACGA

>RPB2 *Fusarium sulawense* NJC33
GCCACCACCAGATCCATGGAGTTCCTCAAGTTCCGTGAATTGCCTGCTGGTCAGAACGCC
ATTGTTGCTATCGCTTGTTATTCAGGATACAACCAGGAAGATTCCGTCATTATGAACCAG
AGCAGTATTGACCGAGGCCTGTTCCGCAGTCTCTTCTTCCGATCATACTCGGATCAGGAG
AAGAAGGTCGGTCTAAACTACACAGAAATCTTCGAGAAGCCCTTCCAACAAACAACGCTT
CGAATGAAGCATGGAACATACGACAAGCTCGACGAGGATGGTATTGTGGCTCCTGGTGTG
CGAGTGTCAGGTGAAGATATCATCATTGGCAAGACTGCGCCTATCGACCAAGAGAATCAA
GATCTCGGTACCAGAACTCAGTCGCACCAGCGCCGCGATATCTCTACACCTCTGCGAAGT
ACAGAGAACGGTATTGTTGATCAAGTCATTTTGACCGTCAACGCCGACAATGTCAAATAC
GTCAAGGTCCGAGTACGAACAACCAAGATCCCCCAGATTGGTGACAAGTTTGCTTCTCGT
CACGGTCAAAAGGGTACAATCGGTGTAACTTACCGACAGGAGGATATGCCCTTCAGCAGA
GAGGGTCTGACTCCCGATATTATTATCAACCCTCACGCCATTCCATCTCGAATGACAATT
GCCCATTTGATTGAGTGTCTGCTAAGTAAAGTCTCAACACTTGAGGGTATGGAGGGTGAC
GCGACGCCCTTTACCGATGTCACTGTCGATTCCGTGTCGGAACTTCTGAGAAAGCACGGC
TACCAGTCTCGAGGTTTCGAGGTTATGTACAATGGTCACACTGGACGTAAGCTCCGCGCC
CAAGTCTTCTTTGGACCTACATACTACCAGCGACTTCGTCACATGG
